# Summary of the Transactions of the College of Physicians of Philadelphia, from November, 1844, to March, 1845

**Published:** 1845-07

**Authors:** 


*Summary of the Transactions of the College of Physicians of Philadelphia, from November*, 1844, *to March*, 1845.

This summary consists of 50 well printed 8vo pages, and contains the Annual Report on Surgery, in addition to much valuable general and statistical information, contained in the reports of the committees on various subjects, and papers read by individual members. It is valuable as the organ of one of the most authoritative medical bodies in existence, and from the fact that its reports are submitted by gentlemen long acknowledged as among the most eminent in our country. We have merely room for a brief analysis of its contents.

At the stated meeting, on Nov. 5th, 1844, the *Annual Report on Surgery* was presented and read by Dr. Parrish. This report contains a tabular summary of the cases of gunshot wounds admitted into the Penn. Hospital, during the riots of May and July, 1844. The table was furnished by Dr. Logan, one of the resident physicians of the Hospital. Of fourteen cases admitted, seven died. "The larger number were admitted on the evening of the seventh of July, during the battle between the mob and the soldiery which occurred in Southwark; and from the contiguity of the Hospital to the scene of action, but little time elapsed before they were placed under surgical treatment. In several of the cases seen by your reporter, where the wounds were mortal, the patients were tormented with that intense and insatiable thirst which occurs in some forms of low fever, and in very prostrate conditions of the system, and which is noticed as among the most horrible torments on the field of battle; together with vomiting, and extreme jactitation and restlessness. In two of the cases, death occurred without reaction, while in several others, the patients lingered in a hopeless condition for several days. It was also remarked that, the wounds by slugs were more severe and dangerous than those by balls—the slug being irregular in shape, and producing more extensive laceration of the parts with which it comes in contact."

Several cases are mentioned in the report of particular interest. In one case, a ball traversed the lower part of the abdomen, without inflicting injury upon any of the viscera; the patient lived fifteen days with the ball lying in the cavity of the abdomen. There were two cases in which the cavity of the chest was penetrated, both of which proved fatal. But one case required amputation. In this case there was a comminuted fracture of the neck and head of the humerus, produced by a grape shot. Amputation at the shoulder joint was performed by Dr. Norris, fifteen hours after the reception of the injury. The operation was performed July 7th, and the patient discharged cured August 20th. In a case of comminuted fracture of the femur, produced by a musket ball, contrary to the weight of authority which demands immediate amputation, an attempt was made to save the limb. The report says of this case: "His youth, temperate habits, and good constitution, were all in his favor, and rendered the case more hopeful than usual. The result appears likely to to meet their most sanguine anticipations, and, should he recover with a good limb, his case will furnish an important addition to our experience on this interesting subject. The result is perhaps mainly attributable to his youth, as it is found that nearly all the cases of recovery after compound fracture of the thigh, whether produced by ordinary accidents or by fire arms, are in persons under age."

Several interesting cases occurring during the riots, and under private treatment, are contained in the report. A case of comminuted fracture of the humerus, with extensive laceration of the soft parts, was successfully treated by Dr. Norris, by amputation near the shoulder joint. A case which fell under the joint care of Drs. Parrish and Remington "is especially worthy of record, from the fact of the perfect restoration of a limb after a pistol shot, which penetrated the thigh, and fractured the femur above the condyles." The ball was smooth, and not larger than large buck shot; "the patient young, of remarkably fine constitution and temperate habits." A case was treated by Dr. Ashmead, of fracture of the patella into several pieces, with opening of the knee joint. The straight splint was used, dressings of lint, careful avoidance of access of air to the knee joint, perfect rest, and opium, stimuli and nutriment to obviate threatened tetanus. At the end of four weeks, the patient "was able to sit up, and was allowed to give slight motion to the joint. When last seen, Nov. 1st, he was found to have as free use of the limb, as is usual after ordinary fracture of the patella—a firm ligamentous union having taken place between the fragments. The circuitous course of the ball, passing around the head of the tibia, and traversing a route of four or five inches without entering the joint, was remarkable—as was the recovery of the use of the limb to its present condition, without serious inflammation and stiffening of the joint."

Other interesting cases are reported, which we have no room to notice.

At the stated meeting of Feb. 4th, 1845, Dr. Moore presented and read the Annual Report on Meteorology and Epidemics. This Report is worthy of much credit, as the means afforded for its preparation in the city of Philadelphia, by the well conducted Dispensaries, the excellent Hospitals, and the Books of the Board of Health, are ample and doubtless correct. We give space to a few extracts from the remarks upon *Prevailing Diseases*.

Of the diseases of the respiratory passages, one third might be referred to the inflammation of the mucous membrane, "constituting the Catarrh and Bronchitis of medical writers. By the latter title, the fatal instances are recorded in the list of interments in the city and adjoining districts. In the period embraced in the present report, such affections have not been considered epidemic. Until after the autumnal equinox the assemblage of symptoms corresponded with the character given to catarrh by nosological writers. In October and the two succeeding months the disease became more prevalent, and was generally ushered in by rigors, accompanied, often, by severe pain in the limbs. To these were superadded great disturbance of the stomach, indicated by nausea and bilious vomiting. In a few cases, diarrhæa was observed. The disturbance of the digestive organs prompted the exhibition of emetics; the operation of which, in many cases, was followed by a cessation of the nausea, and a mitigation of all the symptoms.

"Inflammation of the other pulmonary tissues seems to have been in the proportion and degree observed in ordinary years. The bills of mortality do not indicate any great number of fatal cases..

"Of the Exanthemata, Scarlatina occupied the most conspicuous rank. During the months of January, February, and March, there were many fatal cases. In a widely extended population, varying in habits and constitution, and operated on by causes not yet appreciated by medical writers, Scarlatina, like other epidemics, does not affect different individuals with equal severity. The disease was often mild, consisting merely of a scarlet efflorescence, with little or no affection of the throat. Considerable tumefaction of the tonsils, attended by an accumulation of mucus, and great difficulty in swallowing, was observed in most of the fatal cases. In some instances, extreme prostration of the system was observed at the very onset; in other instances, the indications of danger came on later. Fœtor of the breath was observed in the malignant forms, and was always a cause for apprehending a fatal issue. Death seemed to be often occasioned by the disease pervading the pulmonary tissues, oppressing respiration, and causing a livid appearance of the face. Lethargy, coma, and convulsions, showed that the brain was implicated and death was often induced by the determination or translation of the disease to the cerebral system. On a retrocession of the eruption, the symptoms generally announced this important organ to be affected. The acid odor of the breath was not uniformly associated with the corresponding acid condition of the urine, as observed in former years. The mortality falls far short of what was recorded in 1843.

"To the quality and quantity of the ingesta, many of the acute morbid derangements of the stomach and bowels may be readily traced. Accordingly, affections of this character occur at all seasons of the year. Inflammation of the alimentary canal proceeds more frequently from the food that has been taken, than from any atmospheric influence, though exposure to the cold seems often to contribute in occasioning the disease. The weekly lists of interments present the greatest number of deaths from inflammation of the stomach and bowels in June, and the smallest in February. Colic being caused, most commonly, by irregularities in diet, and by inattention to the due discharge of the fæces, is confined to no period of the year. In September, and during the subsequent months, the disease was often accompanied by bilious vomiting. In these cases, the mild chloride of mercury, given in grain doses, at short intervals, seemed to exert the most favorable influence; correcting the irritability of the stomach, and bringing about a free discharge from the bowels, ineffectually attempted by enemata and purgatives of a bulky form. Some cases of Cholera were noticed in March, apparently occasioned by the character of the food. The Cholera incident to children during the period of the primary dentition, was observed early in May; and proved fatal to eight persons of this age, as recorded in the bills published by the Board of Health. In July, the deaths from this source amounted to one hundred and sixteen. The aggregate number of children who died of Cholera, during the summer months, is stated to have been two hundred and thirty. In 1843, the annual bill exhibits two hundred and sixty-eight deaths from Cholera Infantum.

"Diarrhea and Dysentery were more frequently the subject of medical attention than in ordinary years. Dysentery was comparatively mild, and yielded readily to gentle purgatives, succeeded by the compound powder of ipecacuanha, combined with the blue mass, or given with the syrup of Tolu, according to the ability of the patient to swallow pills, or to take the medicine in the other form."

At the stated meeting, March 4, 1845, Dr. Condie presented the annual report on Diseases of Children. Dr. C. speaks in terms of the highest commendation of the recent work of MM. Rilliet and Barthez on the Diseases of Children, with some cautionary remarks to practitioners, upon the discrimination to be made between the mass of their cases, occurring among the destitute and in hospitals, and those found among the better class in private practice. The report is founded upon the various recent monographs upon the diseases incident to childhood, and their pathology, and contains an excellent and convenient summary of the main facts contained therein.

Much other matter of importance in the pamphlet we must pass over in silence. There is added to the work a Bibliographical Memoir of John C. Otto, M.D., late Vice President of the College of Physicians, read before the College by appointment, March 4th, 1845, by Isaac Parrish, M.D. This is a proper tribute to the memory of an excellent man, an eminent physician, and one of the founders of the College.—Ed.

